# Recrystallization-Induced Surface Cracks of Carbon Ions Irradiated 6H-SiC after Annealing

**DOI:** 10.3390/ma10111231

**Published:** 2017-10-25

**Authors:** Chao Ye, Guang Ran, Wei Zhou, Qiang Shen, Qijie Feng, Jianxin Lin

**Affiliations:** 1 College of Energy, Xiamen University, Xiamen 361102, Fujian, China; kim.yc@foxmail.com (C.Y.); shenqiang1989@126.com (Q.S.); jxlin@stu.xmu.edu.cn (J.L.); 2 China Academy of Engineering Physics, Mianyang 621900, Sichuan, China; zhouwei_801202@163.com (W.Z.); fqj200486@126.com (Q.F.)

**Keywords:** SiC, ion irradiation, surface crack, microstructure, annealing

## Abstract

Single crystal 6H-SiC wafers with 4° off-axis [0001] orientation were irradiated with carbon ions and then annealed at 900 °C for different time periods. The microstructure and surface morphology of these samples were investigated by grazing incidence X-ray diffraction (GIXRD), scanning electron microscopy (SEM), and transmission electron microscopy (TEM). Ion irradiation induced SiC amorphization, but the surface was smooth and did not have special structures. During the annealing process, the amorphous SiC was recrystallized to form columnar crystals that had a large amount of twin structures. The longer the annealing time was, the greater the amount of recrystallized SiC would be. The recrystallization volume fraction was accorded with the law of the Johnson–Mehl–Avrami equation. The surface morphology consisted of tiny pieces with an average width of approximately 30 nm in the annealed SiC. The volume shrinkage of irradiated SiC layer and the anisotropy of newly born crystals during annealing process produced internal stress and then induced not only a large number of dislocation walls in the non-irradiated layer but also the initiation and propagation of the cracks. The direction of dislocation walls was perpendicular to the growth direction of the columnar crystal. The longer the annealing time was, the larger the length and width of the formed crack would be. A quantitative model of the crack growth was provided to calculate the length and width of the cracks at a given annealing time.

## 1. Introduction

SiC can be used as semiconductor devices, electronic devices, optical devices, and sensors based on its unique physical and chemical properties including a wide band gap, high thermal conductivity, stable mechanical properties, and large saturation drift velocity [1,2,3,4]. SiC is also one of the main candidates used as nuclear fuel cladding material and supporting shell in tri-structural isotropic (TRISO) fuel due to its perfect irradiation stability [5,6,7]. Ion implantation is a kind of great method to dope impurities in SiC microelectronic devices [8], but high dose energetic ions could result in SiC amorphization at room temperature [9,10]. After thermal treatment, amorphous SiC would be recrystallized again [11,12,13]. The epitaxial regrowth of different orientation SiC crystals in the ion-irradiation-induced amorphous layer had been widely investigated [14,15,16,17,18]. However, the surface morphology of ion-irradiated SiC could be changed with many surface features appearing after thermal annealing [19,20]. Surface cracking is one of these features, which has a significant influence on the properties of SiC [21]. In fact, surface cracks can be generated on the surface of lots of ion-irradiated solid materials after annealing process [22,23,24]. The main reason for the formation of surface cracks should be attributed to the internal excessive stress including thermal stress [24,25], fatigue stress [26,27], and tensile stress [28,29]. However, the questions are how the stress causes the formation of the cracks in the ion irradiated SiC surface and how the stress is produced during annealing process, which are currently unknown.

There were studies that worked on the relationship between recrystallization process and surface cracks in the irradiated SiC. After absorbing neutrons, the ^30^Si transforms into ^31^Si and then decays to ^31^P atoms [30], which produce a lot of extra carbon atoms. These extra carbon atoms may have a large effect on the SiC properties and performance, but few studies reported the influence of excess carbon on the surface morphology and the microstructure of C^+^ ion irradiated SiC. 

Therefore, in the present work, carbon ions were implanted into single crystal 6H-SiC to simulate the case that extra carbon atoms exist in the SiC and then the evolution of the internal microstructure and surface morphology of SiC after annealing was observed to research the formation mechanism of surface cracks. The type of stress that resulted in formation of cracks and the relationship between recrystallization behavior in the irradiated layer and the formation of surface cracks were determined.

## 2. Experiments

**Figure 1:**
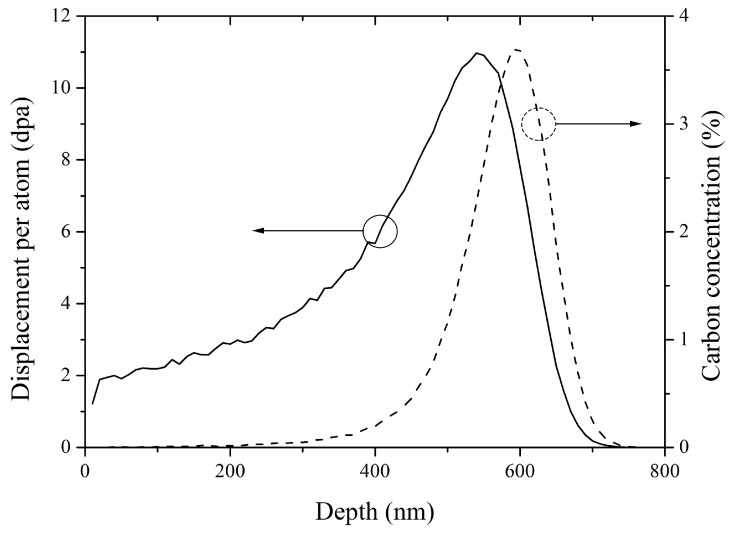
The distribution characteristics of irradiation damage and carbon concentration after 400 keV C^+^ ion irradiation with a fluene of 5 × 10^16^ C^+^/cm^2^ simulated by SRIM-2008 software with quick mode.

Single crystal 6H-SiC wafers with 4° off-axis [0001] orientation were used as original materials in the present work. Ion-irradiation experiments were performed by 400 keV C^+^ ions with a fluence of 5 × 10^16^ C^+^/cm^2^ at room temperature using NEC 400 kV ion implanter in the College of Energy at Xiamen University. We used the Stopping and Range of Ions in Matter (SRIM) software with quick mode to simulate the distribution characteristics of irradiation damage and ion concentration after C^+^ ion implantation. The simulation results were shown in Figure 1. It can be seen that the carbon concentration peak (3.75%) and irradiation damage peak (11 dpa, displacement per atom) appear at the depth of 600 nm and 550 nm, respectively. After C^+^ ion implantation, the samples were annealed at the designed experiment conditions as listed in Table 1 in a tube furnace with argon (99.999% purity) protection. One of the applications of SiC materials was its use as the structural component of TRISO fuel cladding in high temperature gas cooled reactors (HTGCR). The operating temperature ranged from 850 °C to 1000 °C. Therefore, 900 °C was chosen to be the annealing temperature in the present work.

The morphology and topography of the irradiated and then annealed SiC surface were observed by scanning electron microscopy (SEM) (ZEISSEVO18, ZEISS, Heidenheim, Germany). The microstructural phase in the C^+^ ion irradiated layer was characterized by grazing incidence X-ray diffraction (GIXRD) with a fixed angle 1° on a Rigaku D/max-3C X-ray diffractometer (Tokyo, Japan) with CuK_α_ radiation (*λ* = 0.1540598 nm). Under the condition of this fixed incidence angle, the X-ray detection depth is approximately 250 nm, which always locates in the range of the irradiated region according to SRIM simulation results. Transmission electron microscopy (TEM) was used to analyze the microstructure of the irradiated layer. The phase structure in the irradiated layer was characterized using the selected area electron diffraction (SAED) patterns.

Cross-sectional TEM samples prepared by a method of mechanical thinning and then ion milling were used to analyze the microstructure of irradiated layer in a JEOL 2100 transmission electron microscope (JEOL, Tokyo, Japan). TEM samples were first cut from SiC bulks along ion incidence direction and then mechanically polished to approximately 5 μm thickness using diamond sandpapers. Next, the sample was glued on a copper grid by G-1 epoxy glue and finally thinned to approximately 100 nm thickness via Ar^+^ ion milling in the Gatan 695 precision ion polishing system instrument (Gatan, Inc., Pleasanton, CA, USA). 

## 3. Results and Discussion

**Figure 2:**
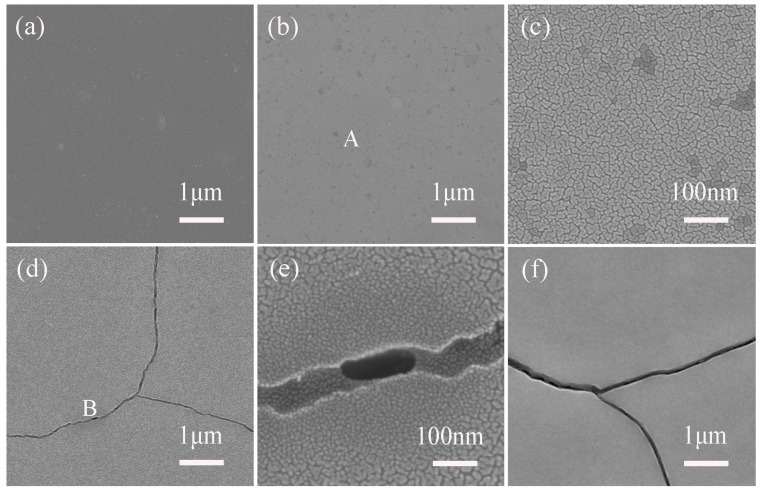
SEM images showing the surface morphology of the as-irradiated (**a**) and then annealed at 900 °C for 0.5 h (**b**), 2 h (**d**), and 10 h (**f**) 6H-SiC samples; (**c**) and (**e**) high magnification images of the characteristic area ‘A’ and ‘B’ in (**b**) and (**d**), respectively.

Figure 2 shows SEM images of the surface morphology of as-irradiated sample and the irradiated samples that were further annealed at 900 °C for 0.5 to 10 h. 

It can be seen that the as-received sample surface is smooth and does not have a special structure as shown in Figure 2a. However, after being annealed for 0.5 h, there are some black spots on the surface. The high magnification of the characteristic region marked by letter ‘A’ in Figure 2b shows that the surface morphology is consisted of tiny pieces with an average width of approximately 30 nm as shown in Figure 2c. When the annealing time is added to 2 h, the cracks with the width of approximately 50 nm appeared throughout the sample surface can be obviously observed as shown in Figure 2d. The high magnification of the feature region marked letter ‘B’ indicates that the pieces located at the edge of the cracks are finer than other surface regions because the tensile stress near the cracking region was larger than that in the other surface regions as shown in Figure 2e. When the annealing time is further increased to 10 h, the width of the cracks increases much more and extends to approximately 200 nm. Except for these SEM images shown here, the surface morphology of non-irradiated samples annealed at 900 °C for 10 h was also observed. Compared with irradiated samples, the non-irradiated samples do not have cracks on their surface after being annealed, which indicates that the above-mentioned changes of surface morphology are not caused by the original substrate. 

**Figure 3:**
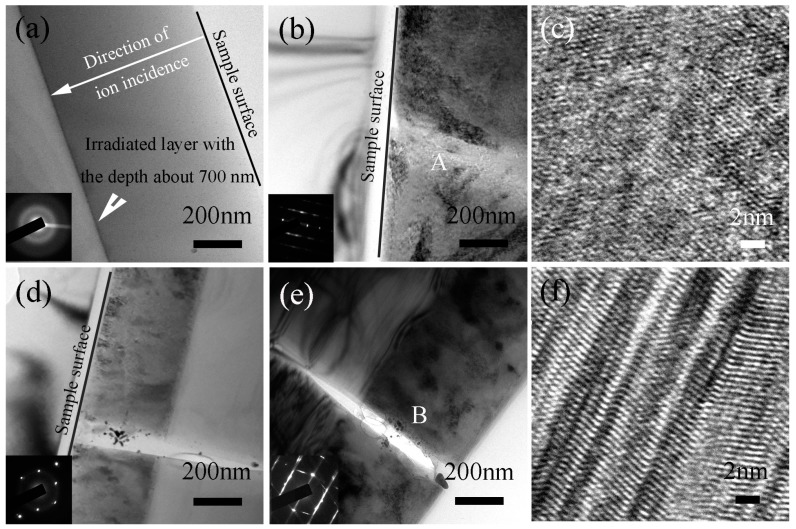
Bright field TEM images of the as-irradiated (**a**) and then annealed at 900 °C for 0.5 h (**b**), 5 h (**d**) and 10 h (**e**) SiC samples. (**c**) and (**f**) HRTEM images of the characteristics regions marked letters ‘A’ and ‘B’ in (**b**) and (**e**), respectively.

Figure 3 is the cross-sectional bright-field TEM images of the as-irradiated sample and the irradiated samples that were further annealed. The direction of ion incidence and irradiated sample surface are marked in TEM images. The SAED pattern of the irradiated area indicates that C^+^ ion irradiation induces the amorphization of SiC matrix as shown in the inserted image at the left bottom in Figure 3a. Matsunaga [31] reported that SiC was changed to amorphization when the irradiation damage was up to approximately 1dpa at room temperature. In the present work, the SRIM simulation results show that the irradiation damage is greater than 1 dpa in the range from the sample surface to the depth of about 700 nm. Therefore, almost the whole irradiated region is amorphous. Bright field TEM image of the irradiated sample annealed at 900 °C for 0.5 h is shown in Figure 3b, which presents a crack initiated in the irradiated region marked letter ‘A’. A high resolution transmission electron microscopy (HRTEM) image of the characteristic region marked letter ‘A’ in Figure 3b is shown in Figure 3c. It can be seen that the annealing induces partially recrystallization in the damaged area. It can also be seen that the irradiated layer is constituted of polycrystalline but no longer single crystal SiC after the annealing process, which could provide a basis for the initiation of cracks at polycrystalline boundaries. After further extending the annealing time, the crack will grow to a large size. The length of the crack is up to approximately 1.3 μm after annealing for 5 h at 900 °C as shown in Figure 3d. The crack not only penetrates to the irradiation region but also propagates into the non-irradiated SiC matrix. Figure 3d shows that the thickness of the damage layer is decreased to approximately 640 nm. The density of SiC in theory will be decreased from (3.21 ± 0.04) g·cm^−3^ to (2.85 ± 0.05) g·cm^−3^ after C^+^ ion irradiation because the crystal SiC is changed completely to amorphous SiC [13]. During the annealing process, the density of irradiated SiC increases when the amorphized SiC gradually transforms to crystal SiC. Simultaneously, the volume of irradiated SiC decreases during the annealing process [13]. Therefore, in our experiment, the decrease of volume can be represented by the decrease of the irradiated layer thickness. After annealing for 10 h, the length and width of the crack become much larger, and the thickness of the irradiated layer is decreased to about 610 nm as show in Figure 3e. HRTEM image of the characteristic region marked letter ‘B’ in Figure 3e indicates that the irradiated area is recrystallized completely after annealing for 10 h at 900 °C as shown in Figure 3f. A large amount of twin structures can be observed in SiC crystal.

**Figure 4:**
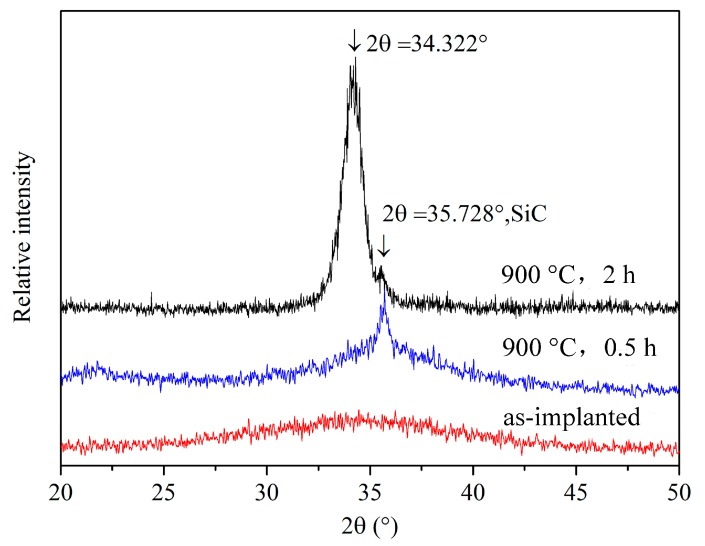
XRD patterns of the as-irradiated SiC sample and the irradiated samples annealed at 900 °C for 0.5 h and 2 h.

Figure 4 shows the GIXRD patterns of the as-irradiated SiC sample and the irradiated samples annealed at 900 °C for 0.5 h and 2 h. It can be seen that there is only an amorphous diffraction hill of the C^+^ ion irradiated sample, which is in accordance with TEM observation results as shown in Figure 3a. When the GIXRD angle of 1° is used, the analyzed depth is approximately 250 nm that locates at the C^+^ irradiated region. After annealing for 0.5 h, a peak of crystal SiC with low diffraction strength located at 2*θ* = 35.728° can be observed in the GIXRD spectrum. At this time, the amorphous phase is still in the majority at the irradiated region. However, when the annealing time is up to 2 h, the diffraction strength of crystal SiC is increased much more. Meanwhile, the location of diffraction peak of crystal SiC shifts a small angle to 2*θ* = 34.322°. The decrease of diffraction angle means the increase of lattice constant of crystal SiC according to Bragg equation, which should be attributed to the increase of the tensile stress in the crystal structure induced by the volume shrinkage during the annealing process. In fact, the larger-sized atoms dissolved into SiC structure will also induce the diffraction angle shift to a low value, but there is no other atoms except for Si and C atoms in the irradiated SiC. Therefore, the volume shrinkage induced stress should be a main reason that causes the GIXRD peak shift to a low angle. During the recrystallization of the irradiation-induced amorphization, the anisotropy of newly born crystal will cause the different shrinkage of each crystal and further induce macroscopic stress difference in a different direction. So in this work, the cracks were formed by tensile stress. However, if it was caused by compressive stress, the diffraction peak of newly born SiC crystal phase should move toward a high angle. 

**Figure 5:**
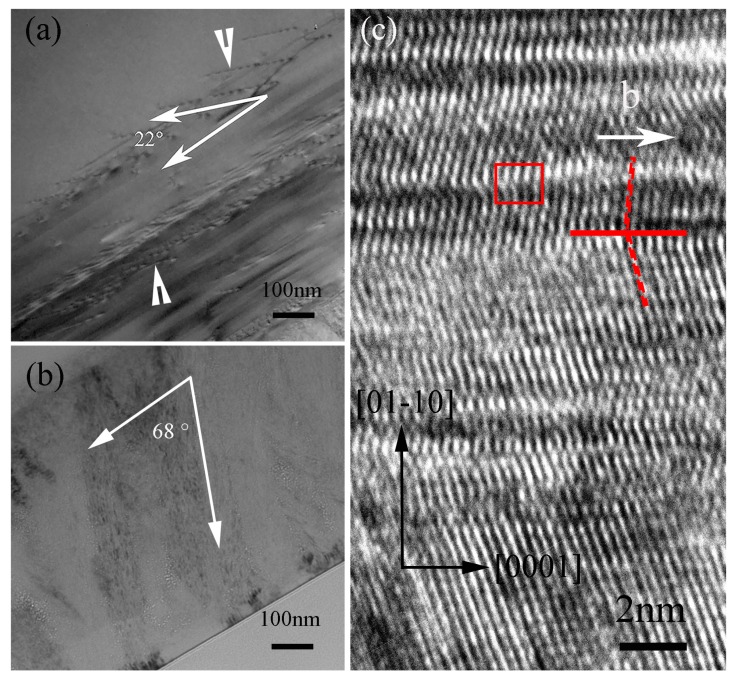
Bright field TEM images of the irradiated samples annealed at 900 °C for 0.5 h (**a**) and 2 h (**b**); (**c**) HRTEM image of the irradiated sample annealed at 900 °C for 10 h.

After being annealed at 900 °C for 0.5 h, a mass of dislocation walls with the angle of 22° to the sample surface appear at a region from the bottom of the irradiated layer to the depth of approximately 1.8 μm, which are marked by white arrows in Figure 5a. According to SRIM simulation results, in fact, there should be no dislocations in the region deeper than the irradiated layer of the single crystal 6H-SiC. The formed dislocation walls should be due to the shrinkage of the irradiated layer during the annealing process as the shrinkage-induced tensile stress also affects the non-irradiated layer. After further being annealed for 2 h. the recrystallization SiC in the form of columnar crystal form in the irradiated layer. The growth direction of the columnar crystals is about 68° to the irradiation surface. Because of the 6H-SiC wafers with 4° off-axis [0001] orientation used in the present wok, the angle between the *c* axe of 6H-SiC and the newly born columnar crystal SiC is actually 72° to the surface plane. Comparing Figure 5a with Figure 5b, it can be seen that the direction of the dislocation walls is perpendicular to the growth direction of the columnar crystal. Figure 5c is HRTEM image of the C^+^ ion irradiated sample annealed at 900 °C for 10 h. The irradiation-induced amorphization almost recrystallizes completely. Many twin planes can be observed in the newly born columnar crystal SiC. One of which is indicated by the red line. Meanwhile, a dislocation indicated by a red square is also shown in Figure 5c, and its Burgers vector is parallel to the crystal orientation. The density of newly born SiC crystal is larger than that of the irradiation-induced amorphous SiC, which induces the shrinkage of volume and further causes a large tensile stress in the recrystallized SiC phase. This process has a great effect on the microstructure and leads to the initiation and propagation of the cracks in the recrystallized SiC.

The characteristic parameter values such as length and width of the cracks, depth of damage layer (DDL), and recrystallization volume fraction (RVF) are measured in the irradiated and annealed SiC and listed in Table 2, which are obtained from the taken SEM and TEM images. The data in Table 2 is an average value coming from 10 measured values. During the characteristic analysis of the RVF, the corresponding DDL is used. For the completely amorphous SiC, the RVF is equal to zero and the DDL is equal to 700 nm. For the complete recrystallization of the irradiation-induced amorphous SiC, the RVF is equal to 1 and the DDL is equal to 610 nm. Under other conditions, the value of RVF is equal to (700-DDL)/(700-610).

With the continuation of the annealing process, the internal shrinkage in the irradiated SiC layer produces a downward tensile stress against the sample surface. When the tensile stress exceeds the fracture stress of the SiC sample, the crack initiates. Because the thickness of the recrystallized layer is very small, it is difficult to accurately measure the internal stress in the newly born SiC and establish the quantitative relationship between the internal stress and the crack length. However, in here, an evolution model between the recrystallization and the surface cracks is provided. The process can be divided into two stages: (i) crack initiation stage and (ii) crack propagation stage.

**Figure 6:**
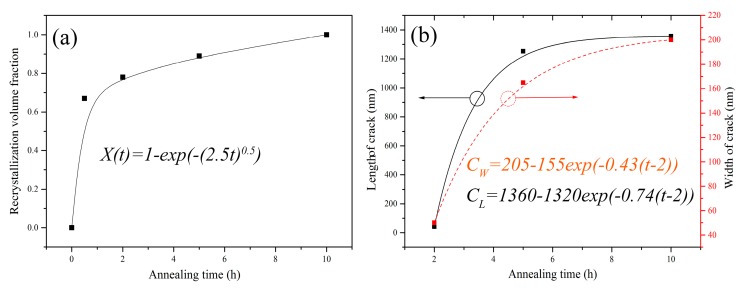
Curves of the characteristic parameters in Table 2 vs. annealing time, (**a**) Recrystallization volume fraction vs. annealing time; (**b**) Length of crack vs. annealing time.

(i) Crack initiation stage. There are two evolution processes in the damaged layer including [13]: (1) Densification caused only by defect annealing; (2) Densification caused by defect annealing and recrystallization. When the recrystallization-induced tensile stress exceeds the fracture stress of the SiC sample, the crack will initiate. The time of first process is very short and the corresponding effect can be ignored. For the second process, the Johnson–Mehl–Avrami equation can be used to describe the densification as shown in the equation

(1) $$X\left(t\right)=1-exp\left(-{\left(Kt\right)}^{n}\right)$$

where *X(t)* is the RVF at a given temperature, *t* is the time, *n* is Avrami index that is related to the phase transition mechanism and is generally in the range of 1 to 4; *K* is a constant. The Johnson–Mehl–Avrami equation is assumed that the nucleation process is a homogeneous process. Through the curve fitting of the data in Table 2, *K* is 2.5, and *n* is 0.5 in the present work as shown in Figure 6a. It can be seen that *n* is not located in the range from 1 to 4, which could be attributed to the inhomogeneous recrystallization process because of C^+^ ion irradiation. Therefore, it needs to modify the parameter *n* for different samples.

(ii) Crack propagation stage. From TEM and SEM images, the cracks have already actually initiated while the recrystallization process has not be completely done, for example in the irradiated SiC annealed for 2 h at 900 °C. According to other relevant studies [22,23,24] and our current research results, the crack propagation should be caused by the tensile stress produced by the annealing-induced recrystallization. A probabilistic crack model with a power law was defined and developed by P. Rajeev and Tesfamariam [32] as shown in the equation

(2) $$C_{W}=a\times \Delta T^{b}$$

where *C_W_* is the crack width; ∆*T* is a temperature difference between the initial temperature and annealing temperature; *a* and *b* are fitting coefficients according to the experiment data. We try to replace the temperature variation ∆*T* with the time variation ∆*t* to fit the experiment results, but the effect is not optimistic. Therefore, considering the power law model and the actual experiment results, a crack propagation model is provided as

(3) $$C=C_{max}-Ae^{-B\left(t-t_{0}\right)}$$

where *C* is the width (or length) of crack at a specific experiment condition, *C_max_* is the maximum value of crack parameters (width or length) at the end of recrystallization process, *t* is annealing time; *t_0_* is an exact time that should be the time of crack initiation in theory; *A* and *B* are constants that are correlated with the type of sample, annealing temperature, species, and fluence of the implanted ions. In the present work, we assume that the time of crack initiation is approximately 2 h of annealing. The best fitting results shown in Figure 6b are that *C_max.L_* is 1360; *A_L_* is 1320; *B_L_* is 0.74; *t*_0*.L*_ is 2; *C_max.W_* is 205; *A_W_* is 155; *B_W_* is 0.43 and *t*_0*.W*_ is 2 (Subscript *L* and *W* denote the length and width of the formed crack, respectively). The equation of crack propagation is shown as

(4) $$C_{W}=205-155e^{-0.43\left(t-2\right)}$$

(5) $$C_{L}=1360-1320e^{-0.74\left(t-2\right)}$$

Therefore, for C^+^ ion irradiated 6H-SiC, after being annealed at 900°C, the length and width of the formed crack can be obtained in any annealing time according to the Equations (4) and (5). 

## 4. Conclusions

Single crystal 6H-SiC wafers with 4° off-axis [0001] orientation were irradiated with carbon ions and then annealed at 900 °C for different time. The microstructure and surface morphology of these samples were investigated by GIXRD, SEM, and TEM. Ion irradiation induced SiC amorphization and the decreased density of the irradiated SiC layer. The surface was smooth and did not have special structure in the as-received sample. While after annealing at 900 °C for 0.5 h, the surface morphology was consisted of tiny pieces with an average width of approximately 30 nm. After annealing at 900 °C, the amorphous SiC were recrystallized to form columnar crystals. A large amount of twin structures were formed in the columnar crystals. The longer the annealing time was, the more the amount of recrystallized SiC would be. The recrystallization volume fraction could be calculated according to the Johnson–Mehl–Avrami equation. The recrystallization induced the increased density and the volume shrinkage of the irradiated SiC layer, which produced the internal stress in the irradiated SiC layer and then caused a large number of dislocation walls in the non-irradiated layer. The direction of dislocation walls was perpendicular to the growth direction of the columnar crystal. When the annealing time was up to a certain value, the cracks initiated in the newly born SiC layer. The longer the annealing time was, the larger the length and width of the formed cracks would be. However, the increment rate of the length and width of the cracks gradually reduced in the form of a negative exponent, and finally reached a stable value. A quantitative model of the crack growth was provided to calculate the length and width of the cracks at a given annealing time.

## Figures and Tables

**Table 1:** Sintering parameters of the C^+^ ion implanted 6H-SiC samples.

Samples	#1	#2	#3	#4	#5
Annealing temperature (°C)	RT	900	900	900	900
Annealing time (h)	0	0.5	2	5	10

**Table 2:** Measurement values of the characteristic parameters in the SiC layer.

Sample	Length of Crack (nm)	Width of Crack (nm)	Depth of Damage Layer (nm)	Recrystallization Volume Fraction
#1	0	0	700	0
#2	0	0	640	0.67
#3	42	50	620	0.88
#4	1253	165	612	0.98
#5	1357	200	610	1
